# Type 2 Innate Lymphoid Cells Accumulate in the Brain After Hypoxia-Ischemia but Do Not Contribute to the Development of Preterm Brain Injury

**DOI:** 10.3389/fncel.2020.00249

**Published:** 2020-08-07

**Authors:** Aura Zelco, Eridan Rocha-Ferreira, Arshed Nazmi, Maryam Ardalan, Tetyana Chumak, Gisela Nilsson, Henrik Hagberg, Carina Mallard, Xiaoyang Wang

**Affiliations:** ^1^Department of Physiology, Institute of Neuroscience and Physiology, Sahlgrenska Academy, University of Gothenburg, Gothenburg, Sweden; ^2^Centre of Perinatal Medicine & Health, Institute of Clinical Sciences, Sahlgrenska Academy, University of Gothenburg, Gothenburg, Sweden; ^3^Henan Key Laboratory of Child Brain Injury, Institute of Neuroscience and Third Affiliated Hospital of Zhengzhou University, Zhengzhou, China

**Keywords:** preterm brain injury, innate lymphoid cells, hypoxia-ischemia, innate immunity, newborns

## Abstract

**Background:**

The immune system of human and mouse neonates is relatively immature. However, innate lymphoid cells (ILCs), commonly divided into the subsets ILC1, ILC2, and ILC3, are already present in the placenta and other fetal compartments and exhibit higher activity than what is seen in adulthood. Recent reports have suggested the potential role of ILCs, especially ILC2s, in spontaneous preterm labor, which is associated with brain damage and subsequent long-term neurodevelopmental deficits. Therefore, we hypothesized that ILCs, and especially ILC2s, play a role in preterm brain injury.

**Methods:**

C57Bl/6J mice at postnatal day 6 were subjected to hypoxia-ischemia (HI) insult induced by left carotid artery ligation and subsequent exposure to 10% oxygen in nitrogen. The presence of ILCs and ILC2s in the brain was examined at different time points after HI. The contribution of ILC2s to HI-induced preterm brain damage was explored using a conditionally targeted ILC2-deficient mouse strain (*Rorα^*fl/fl*^IL7r^*Cre*^*), and gray and white-matter injury were evaluated at 7 days post-HI. The inflammatory response in the injured brain was assessed using immunoassays and immunochemistry staining.

**Results:**

Significant increases in ILCs and ILC2s were observed at 24 h, 3 days, and 7 days post-HI in the injured brain hemisphere compared with the uninjured hemisphere in wild-type mice. ILC2s in the brain were predominantly located in the meninges of the injured ipsilateral hemispheres after HI but not in the brain parenchyma. Overall, we did not observe changes in cytokine/chemokine levels in the brains of *Rorα^*fl/fl*^IL7r^*Cre*^* mice compared with wild type animals apart from IL-13. Gray and white-matter tissue loss in the brain was not affected after HI in *Rorα^*fl/fl*^IL7r^*Cre*^* mice. Correspondingly, we did not find any differences in reactive microglia and astrocyte numbers in the brain in *Rorα^*fl/fl*^IL7r^*Cre*^* mice compared with wild-type mice following HI insult.

**Conclusion:**

After HI, ILCs and ILC2s accumulate in the injured brain hemisphere. However, ILC2s do not contribute to the development of brain damage in this mouse model of preterm brain injury.

## Introduction

Preterm infants are highly susceptible to brain damage, which may lead to long-term neurodevelopmental deficits such as cerebral palsy and cognitive impairments. Therapeutic hypothermia is used for term newborns, and other strategies such as erythropoietin are being tested; however, there are still no effective treatments for brain damage in preterm newborns (Shankaran et al., 2005; Zhu et al., 2009; Shankaran, 2015; Natalucci et al., 2016; Song et al., 2016; Juul et al., 2020). The mechanisms underlying perinatal brain damage are not fully understood, but hypoxia-ischemia (HI) and maternal/neonatal inflammation have been suggested as major etiological factors. Apoptosis, mitochondrial dysfunction, excitotoxicity, and impaired oligodendrocyte maturation are also implicated in the injury process, as is the involvement of immune cells (Yang et al., 2014; Hagberg et al., 2015; Zhang et al., 2017; Albertsson et al., 2018; Herz et al., 2018; Nazmi et al., 2018).

Since their discovery about a decade ago, innate lymphoid cells (ILCs), which are part of the innate immune system, have been studied intensively. ILCs lack antigen recognition and therefore can respond quickly to a variety of stimuli in their immediate surroundings (Spits et al., 2013; Vivier et al., 2018). ILCs are usually divided into subtypes based on the expression of the transcription factors that regulate their development, cytokine production, and function. These cells mirror the T-helper (Th) cells of the adaptive immune system in terms of their cytokine production and functions (Almeida and Belz, 2016). Type 1 ILCs (ILC1s), including natural killer cells, respond similarly to Th1 cells. ILC2s mirror Th2 cells with the production of cytokines such as interleukin (IL)-4, IL-5, and IL-13, which are involved in allergic inflammation. ILC3s are similar to Th17 cells and secrete IL-17 and IL-22 upon activation (Spits et al., 2013; Vivier et al., 2018). The immune response in neonates is biased toward the Th2 type (Adkins et al., 2004; Debock and Flamand, 2014), but after HI insult there is a Th1/Th17-type immune response in the neonatal mouse brain (Albertsson et al., 2014; Yang et al., 2014) and blockage of lymphocyte trafficking to the brain is neuroprotective (Yang et al., 2014). Further, the Th2 cytokines IL-4 and IL-13 have been found to protect the mouse brain from injury (Walsh et al., 2015; Kolosowska et al., 2019).

ILCs have been shown to play an important role in the immune response against viruses, allergens, and lung and intestinal inflammation (Nussbaum et al., 2013; Bernink et al., 2014; Oliphant et al., 2014; Besnard et al., 2015). Furthermore, ILCs develop early in ontogeny and are present already in the placenta and in other fetal compartments like the thymus, the liver, cord blood, and bone marrow (Forsberg et al., 2014; Jones et al., 2018; Miller et al., 2018; Xu et al., 2018) and therefore might be especially important for immune responses in early life. Recent studies have shown that ILC2s are involved in asthma-like responses after neonatal hyperoxia (Cheon et al., 2018) and that together with ILC3s they are increased when spontaneous preterm birth occurs (Xu et al., 2018), suggesting the possible involvement of these cells in injurious events around birth. Additionally, ILCs, in particular ILC2s, have been found in the murine central nervous system (Mair and Becher, 2014; Besnard et al., 2015; Russi et al., 2015; Fung et al., 2020), especially in the meninges (Gadani et al., 2017; Russi et al., 2018), and they are involved in the neuroinflammatory response associated with experimental autoimmune encephalomyelitis (Mair and Becher, 2014), aging (Fung et al., 2020), and cerebral malaria (Besnard et al., 2015). In a mouse model of spinal cord injury, meningeal ILC2s are functionally activated, enter the injury site, and produce type 2 cytokines that up-regulate inflammatory genes and improve recovery, thus suggesting that these cells can also play a beneficial role in preventing CNS injury (Gadani et al., 2017).

Given the early development and functional maturity of ILCs in ontogeny, the rapid response of ILCs to insults in various tissues, and their potential role in cerebral pathologies, we hypothesize that ILCs accumulate in the brain after HI insult and are involved in the injury process in the mouse model of preterm brain injury.

## Materials and Methods

### Experimental Animals

Mice were bred at our animal facility (Experimental Biomedicine, University of Gothenburg), and pups of both sexes were used. C57Bl/6J mice (Charles River, Germany) were used for flow cytometry experiments and meningeal immunofluorescence staining. The transgenic mouse strain *Rorα^*fl/fl*^IL7r^*Cre*^*, that were generated by crossing *Rorα^*fl/fl*^* mice and *IL7-Cre* mice, was a kind gift from Professor Andrew McKenzie (Cambridge University, United Kingdom). The floxed gene was retinoid-related orphan receptor alpha (RORα), a transcription factor that is critical for ILC2 development. The Cre-recombinase enzyme was linked to interleukin-7 receptor (IL7R), a transmembrane receptor restricted to the lymphoid lineage. This combination leads to impairment of ILC2s, as described previously (Oliphant et al., 2014). *Rorα^*fl/fl*^IL7r^+/*Cre*^* (ILC2-impaired) and *Rorα^*fl/fl*^IL7r^+/+^* (wild-type controls) mouse littermates were used in the experiments. These mice were phenotypically indistinguishable from commercially available C57Bl/6J mice. Genotyping of *Rorα^*fl/fl*^* mice was undertaken using PCR primers (5′ TGA GTG GTA ACA CCA CGG CAC GC 3′ and 5′ TGG AGC AGA ATC ATC CAG GAG GCC 3′), giving a wild-type product of 573 bp and a targeted product of ∼650 bp. For genotyping of *IL7-Cre* mice, the PCR primers were 5′ CCT GAA AAC TTT GCC CCC TCC ATA 3′, 5′ CCA TAG AAT AGT GCA GCC TTG CCT C 3′, and 5′ AGC GAA AGC TCT ACC CAG AGC 3′, and these generated a wild-type product of 584 bp and a targeted product of 680 bp.

**FIGURE 1:**
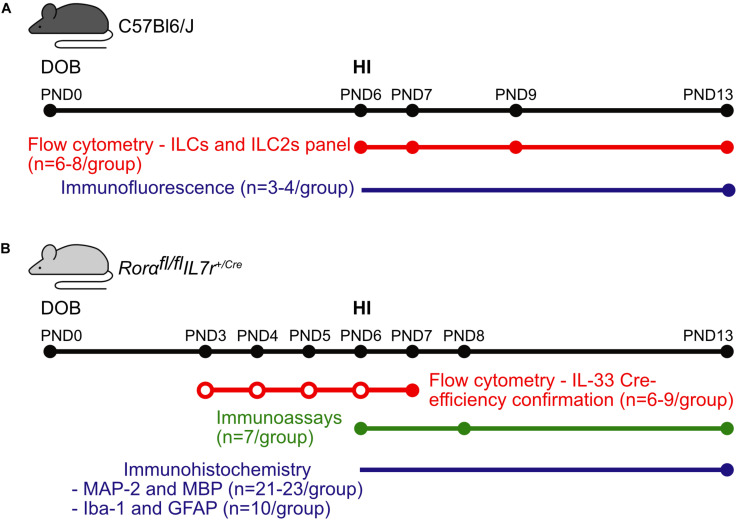
Graphical abstract of the experiments. Representative scheme of the methods, numbers of animals, and time points used for experiments in **(A)** the C57Bl6/J mouse strain and **(B)** the *Rorα^*fl/fl*^IL7r^*Cre*^* mouse strain. Colored dots correspond the data collection time points for the respective methods, while the empty dots correspond to the IL-33 injections. Abbreviations: DOB: date of birth, PND: postnatal day, IL-33: interleukin-33, HI: hypoxia-ischemia.

The animal facility had a 12-h light-dark cycle, and the mice had free access to standard chow (B&K, Solna, Sweden) and water. All experiments were performed with the approval of the Regional Animal Ethical Committee of Gothenburg (ethical permit numbers 58/2016 and 2042/18). The experimental design and the number of animals used for each experiment are shown in Figure 1.

### Hypoxic-Ischemic Model

The day of birth was defined as postnatal day (PND)0. At PND6, HI surgery was performed as described previously (Albertsson et al., 2014). The pups were anesthetized with isoflurane (5% for induction and 3% for maintenance) and underwent unilateral left carotid artery ligation followed by xylocaine as the local anesthetic. After recovering with their dam for 1 h, the pups were transferred to a humidified chamber for exposure to 70 min of hypoxia (10% O_2_ in N_2_, 36.0 ± 0.5°C) (Rice et al., 1981). Before and after the hypoxia, the pups rested for 10 min at 36.0 ± 0.5°C in the chamber. The pups were then returned to the dam’s cage until being sacrificed.

### IL-33 Stimulation of ILC2 Expansion

To test the Cre efficiency in neonatal ILC2-impaired mice, we challenged wild type and ILC2-impaired pups with IL-33, which promotes ILC2 expansion (Oliphant et al., 2014). Briefly, the pups received intraperitoneal (i.p.) injections of either mouse recombinant IL-33 (0.04 μg/μl/g body weight in PBS, BioLegend) or PBS alone (*n* = 6–9/group) every 24 h from PND3 to PND6 for a total of four injections. The pups were sacrificed 24 h after the last injection (PND7), and their lungs were collected for flow cytometry analysis to determine the number of IL-33–induced ILC2s.

### Flow Cytometry Experiments

Flow cytometry was used to investigate the presence of all ILCs in the brains of C57Bl/6J mice at 6 h, 24 h, 3 days, and 7 days after HI with naïve littermates as controls (*n* = 6–8/group). Transcardial saline perfusion was performed after i.p. injection of pentobarbital (50 mg/ml, Abcur AB, Helsingborg, Sweden). The brains were dissected out, the cerebellum was removed, and the ipsilateral and contralateral hemispheres, including the meninges, were processed separately to obtain single-cell suspensions as described previously (Zhang et al., 2017; Nazmi et al., 2018). In brief, brain homogenate samples were incubated with an enzymatic solution composed of 0.01% papain, 0.01% DNase I (Worthington, NJ, United States), 0.1% Dispase II (Roche, Sweden), and 12.4 mM MgSO_4_ in Ca^2+^/Mg^2+^-free HBSS (Thermo Fisher Scientific, Sweden) at 37°C for 20 min. The monocyte population was then separated on a Percoll gradient (30/70%). After blocking non-specific binding with Fc block (C16/32; clone 2.4G2; cat. 553142, BD Pharmingen), the following primary antibodies were used: anti-lineage cocktail (FITC; CD3, CD5, B220, CD11b, CD11c, Gr-1, TCRgd, and Ter-119; cat. 22-7770-72, eBioscience), anti-CD45 (APCcy7; clone 30-F77; cat. 103115, BioLegend) and anti-CD90 (Thy1.2, PEcy7; cat. ABIN477038, eBioscience). ILC2s were identified by the addition of anti-SCA-1 (PE; clone D7; cat. 108107, BioLegend), anti-NKp46 (BV510; clone 29A1.4; cat. 563455; BD Biosciences), anti-KLRG1 (APC; clone 2F1; cat. 561620, BD Biosciences), and anti-GATA3 PE (Catalog # 12-9966-42, eBiosciences) antibodies. For intracellular staining, the permeabilization and fixation procedure was performed according to manufacturer’s protocol. Briefly, after incubation with 250 μl of BD Cytofix/Cytoperm solution, the cells were washed in 1 × BD Perm/Wash buffer (BD Biosciences, CA, United States) and resuspended in 100 μl of 1 × BD Perm/Wash buffer before the GATA3 intracellular staining. The samples were run on a BD FACSCanto II^TM^ flow cytometer. Fluorescence-minus-one controls for each antibody were run together with each experiment. Lung tissues in which ILC2s were enriched were included as positive controls for all brain tissue flow cytometry experiments, and the data were analyzed with the FlowJo v10 software (Tree Star, Ashland, OR, United States).

The lung single-cell suspensions were prepared following a previously described method (Moro et al., 2015) with minor modifications. Briefly, after perfusion the lungs were extracted, minced into small pieces, and then digested for 45 min at 37°C with the same enzyme mix used for the brain dissociation. The supernatant was incubated with Red Blood Cell Lysing Buffer (R7757, Sigma-Aldrich, Sweden) at room temperature. Samples were then stained and analyzed as above.

### Immunoassay for Cytokines and Chemokines

Samples were collected at 6 h, 48 h, and 7 days after HI from wild type and ILC2-impaired mice (*n* = 7/group) for immunoassays of cytokine and chemokine protein expression. In summary, after pentobarbital i.p. injection the animals underwent transcardial saline perfusion. The ipsilateral and contralateral hemispheres, including the meninges, were collected separately and stored at -80°C until processing. The Bio-Plex Pro^TM^ Mouse Cytokine 23-plex Assay (#m60009rdpd, Bio-Rad, Table 1) was used according to the manufacturer’s instructions.

**TABLE 1:** Cytokines and chemokines analyzed with the Bio-Plex Pro^TM^ Mouse Cytokine 23-plex Immunoassay.

**Abbreviation**	**Cytokine/Chemokine**	**Abbreviation**	**Cytokine/Chemokine**
IL-1α	Interleukin 1α	IL-17	Interleukin 17
IL-1β	Interleukin 1β	Eotaxin	Eotaxin
IL-2	Interleukin 2	G-CSF	Granulocyte-colony stimulating factor
IL-3	Interleukin 3	GM-CSF	Granulocyte-macrophage colony-stimulating factor
IL-4	Interleukin 4	IFN-γ	Interferon γ
IL-5	Interleukin 5	KC	Chemokine (C-X-C motif) ligand 1
IL-6	Interleukin 6	MCP-1	Monocyte chemoattractant protein 1
IL-9	Interleukin 9	MIP-1α	Macrophage inflammatory protein 1α
IL-10	Interleukin 10	MIP-1β	Macrophage inflammatory protein 1β
IL-12(p40)	Interleukin 12 p40	RANTES	Regulated on activation, normal T cell expressed and secreted
IL-12(p70)	Interleukin 12 p70	TNF-α	Tumor necrosis factor α
IL-13	Interleukin 13		

### Immunohistochemistry and Immunofluorescence Staining

At PND13 (7 days after HI), wild type and ILC2-impaired mice were deeply anesthetized by i.p. injection with pentobarbital and then perfused-fixed with 5% buffered formaldehyde (Histofix, Histolab, Sweden). For Immunohistochemistry staining, the brains with meninges were collected and stored at 4°C in Histofix before paraffin-embedding and sectioning. Coronal sections of the forebrain were cut with a thickness of 7 μm. To obtain a representation of the areas of interest, the staining was performed for evaluating gray matter on every 50th section (6 sections/animal). For white-matter assessment, and microglial and astrocyte assessments, every 100th section was used (3 sections/animal). Briefly, the sections were de-paraffinized followed by antigen retrieval and 3% H_2_O_2_ in phosphate buffer. After blocking, sections were incubated with mouse anti-microtubule-associated protein 2 (MAP-2, 1:1,000 dilution; M4403, Sigma-Aldrich, United States), mouse anti-myelin basic protein (MBP, 1:1,000 dilution; SMI-94 Covance, United States), rabbit anti-ionized calcium binding adaptor molecule 1 (Iba-1, 1:2000 dilution, 019-19741, FUJIFILM *Wako* Chemicals, United States), and rabbit anti-Glial fibrillary acidic protein (GFAP, 1:500, Z0334, Dako, Denmark) primary antibodies overnight at 4°C. The following day, the sections were incubated with the corresponding secondary antibodies for 1 h at room temperature. The sections were then incubated for 1 h in ABC Elite, and the immunoreactivity was visualized with 0.5 mg/ml 3.3-diaminobenzidine in a buffer consisting of NiSO_4_, β-D-glucose, NH_4_Cl, and β-D-glucose oxidase (all from Sigma-Aldrich, Sweden).

Immunofluorescence was performed to identify ILC2s at 7 days after HI (*n* = 3/group). After saline perfusion, the meninges from each hemisphere were peeled off from the parenchyma and placed separately on a glass slide. Brains and lungs were collected as well and stored in Histofix followed by sucrose 30%. Both tissues were then frozen in isopentane and cut at 30 μm of thickness. After fixation and blocking with 5% donkey serum at room temperature for 1 h, the tissues were incubated with rat anti-mouse CD3 (1:500 dilution, cat. 100202, BioLegend) and rabbit anti-mouse ST2 (1:250 dilution, PA5-20077, Invitrogen) primary antibodies overnight at 4°C. The tissues were then incubated for 1 h at room temperature with donkey anti-rat Alexa Fluor 488^®^ (1:1,500 dilution, A21208, Invitrogen) and donkey anti-rabbit Alexa Fluor 594^®^ (1:1,500 dilution, A21207, Invitrogen) secondary antibodies. Lung tissue was used as the positive control, and omitting the primary antibodies was used as the negative control (Supplementary Figure 1). The slides were then stained with DAPI and afterward mounted with coverslips and ProLong^TM^ gold antifade reagent (P36930, Invitrogen).

### Quantification of Gray and White-Matter Tissue Loss

Gray-matter tissue loss was assessed by measuring MAP-2^+^ areas, either as a percentage of volume (mm^3^) or area (mm^2^) for each stained section at different levels as described previously (Zhang et al., 2017; Albertsson et al., 2018). Images of the stained sections were acquired with an Olympus Optical microscope using a 1.25 × objective lens. MAP-2^+^ areas were delineated using ImageJ software (Rasband, W.S., US National Institutes of Health, United States^1^).

To assess white-matter injury, a newly established automated segmentation method, MyelinQ, was used to measure the size of the MBP^+^ areas (Mottahedin et al., 2018). Briefly, we captured images of each section using a 5 × objective lens with the newCAST software (Visiopharm, Denmark) on a modified Leica microscope (Leica DM6000 B, Germany) equipped with a motorized stage (Ludl MAC 5000, United States) and a digital camera (Leica DFC 295, Germany). MyelinQ was used for automatic detection of MBP^+^ areas in the whole brain hemisphere.

For the quantification of gray and white-matter loss, the sizes of the MAP-2^+^ and MBP^+^ areas in the contralateral and ipsilateral hemispheres were measured separately in order to calculate the tissue damage in the ipsilateral hemisphere against the internal control (the contralateral hemisphere). The total tissue loss was calculated with the following formula (Albertsson et al., 2014):

(contralateral⁢area-ipsilateral⁢area)/contralateral⁢area×100%

The total tissue loss volume was calculated as:

Volume=sum⁢of⁢section⁢area×section⁢thickness×(1/sampling⁢fraction)

### Quantification of Iba-1 Positive Brain Region and GFAP-Positive Astrocytes

The border zone between the injured and non-injured brain areas in the ipsilateral hemispheres, as well as the corresponding brain regions in the contralateral brain hemispheres were selected for analysis. Using ImageJ software (v1.52a, NIH, United States), GFAP-positive astrocytes were counted on captured images with an Olympus Optical microscope using a 40× objective lens from selected areas in two regions of interest per section (in total 6 regions of interest per brain), and expressed as cell density (cells/mm^2^). The Iba-1–positive stained brain regions were measured on the images which were captured by a 20× objective lens from eight regions of interest per brain. Analysis was performed on the images with final resolution of 1360 × 1024 pixel after adjusting threshold of positive stained areas on ImageJ. Data were expressed as the ratio between Iba-1 stained area and total regions of interest.

### Meningeal ILC2 Cell Counting

A systematic set of Z-stacks of three regions of interest per hemisphere were acquired with a 20× objective lens on LSM 800 confocal microscope (Carl Zeiss, Germany). ILC2s (defined as CD3^–^ST2^+^) were blindly counted in the height of the Z (*z* step = 1 μm), and volumes of each regions of interest were calculated. Estimation of number density was performed by applying the following formula (McNaught and Wilkinson, 1997) of paired regions of interests from contralateral and ipsilateral meninges:

N=ΣQ/-V

*N* is the total number of cells per volume of brain region; ΣQ^–^ is the number of counted cells; V is the volume of regions of interest per sampling frame.

### Statistical Analysis

All statistical analyses were performed with IBM SPSS Statistics 25 (IBM Corp, Armonk, NY, United States). Data were tested for normal distribution through the generation of QQ plots, and equality of variance was assessed by Leven’s test. If normally distributed, the data were analyzed with the corresponding parametric test, while in the case when the data were non-normally distributed, appropriate non-parametric tests were applied. All data are presented as boxplots (5th – 95th percentiles). Statistical significance was considered as *p*-values < 0.05.

## Results

### The Frequencies of ILCs and ILC2s Increased in the Neonatal Mouse Brain After HI Injury

**FIGURE 2:**
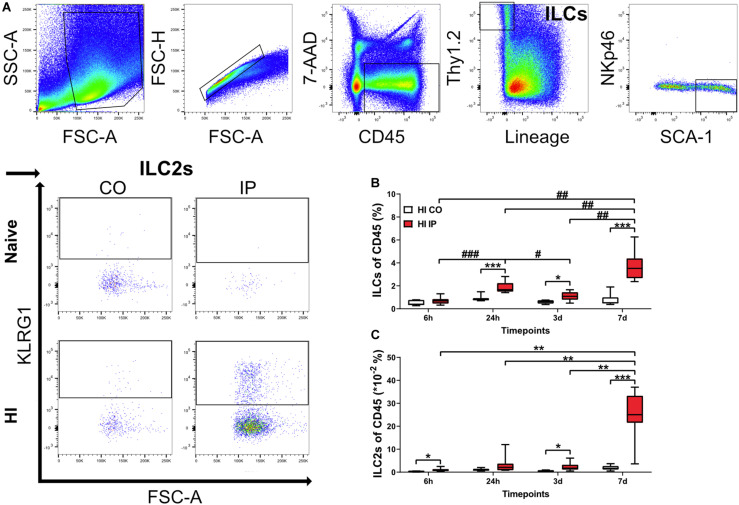
ILCs and ILC2s increased in the ipsilateral hemispheres in the neonatal mouse brain in a time-dependent fashion after HI injury. Single-cell suspensions generated from brain homogenates were analyzed using flow cytometry in mouse pups at different time points after HI, with naïve C57Bl6/J mouse littermates as controls (*n* = 6–8/group). **(A)** Representative flow cytometry plots showing the gating strategy for ILCs and ILC2s. Both are represented as percentages of the CD45^+^ population. ILCs **(B)** and ILC2s **(C)** in the ipsilateral and contralateral hemisphere at 24 h, 3 days, and 7 days after HI. A mixed model ANOVA with Games-Howell post-hoc were used. *#: *p* < 0.05, **##: *p* < 0.01, ***###: *p* < 0.001. in panels (**B,C**). Abbreviations: N: naïve, HI: hypoxia-ischemia, IP: ipsilateral hemisphere, CO: contralateral hemisphere.

ILC2s are the subtype that are most frequently observed in the adult mouse brain compared to ILC1s and ILC3s (Russi et al., 2015; Gadani et al., 2017). To determine if ILCs were present in the neonatal mouse brain, we performed flow cytometry analysis on single-cell suspensions isolated from brain homogenate (Figures 2A–C, Supplementary Figure 2), and measured the frequencies of both ILCs and ILC2s in naïve mice (*n* = 6/time point) and in mice at 6 h (*n* = 8), 24 h (*n* = 7), 3 days (*n* = 8), and 7 days (*n* = 7) after HI. Because of the lack of single specific markers, we selected a panel of surface markers in the FACS analysis for their identification, and ILCs were defined as CD45^+^Lin^–^Thy1.2^+^ (Pelly et al., 2016) and ILC2s were defined as CD45^+^Lin^–^Thy1.2^+^SCA-1^+^NKp46^–^KLRG1^+^ (Cardoso et al., 2017).

For both ILCs and ILC2s, we did not detect any differences between naive and contralateral brain hemispheres at any time points after HI; therefore, data from naïve mice are not shown. The frequencies of ILCs were significantly increased in the brain hemisphere ipsilateral to the injury compared to the uninjured contralateral hemisphere at 24 h (*p <* 0.001), 3 days (*p* = 0.010), and 7 days (*p <* 0.001) after HI, but not at 6 h after HI (Figure 2B). Quantification of ILC2s showed a significant increase in the ipsilateral hemisphere compared with the uninjured contralateral hemisphere at 6 h (*p* = 0.011), 3 days (*p* = 0.020), and 7 days (*p <* 0.001) after HI (Figure 2C).

Furthermore, the frequencies of both ILCs (Figure 2B) and ILC2s (Figure 2C) in the ipsilateral hemisphere of HI mice showed an increasing trend over time, with the highest at 7 days after HI for both populations. We observed a first small peak for ILCs at 24 h, which was higher than 6 h (*p* < 0.001) and the subsequent 3 days (*p* = 0.031) time points. The ILCs showed a second peak and were significantly greater at 7 days compared with 6 h (*p* < 0.001), 24 h (*p* = 0.009), and 3 days (*p* = 0.002) after HI (Figure 2B). ILC2s instead increased drastically only at 7 days compared with 6 h (*p* < 0,001), 24 h (*p* = 0.002), and 3 days (*p* = 0.002) after HI (Figure 2C). The increased ILC2s at 7 days after HI in the ipsilateral hemisphere were further confirmed using intracellular staining for GATA3, the key transcription factor and master regulator that is critical for the development and maintenance of ILC2s (Yagi et al., 2014) (Supplementary Figure 3).

**FIGURE 3:**
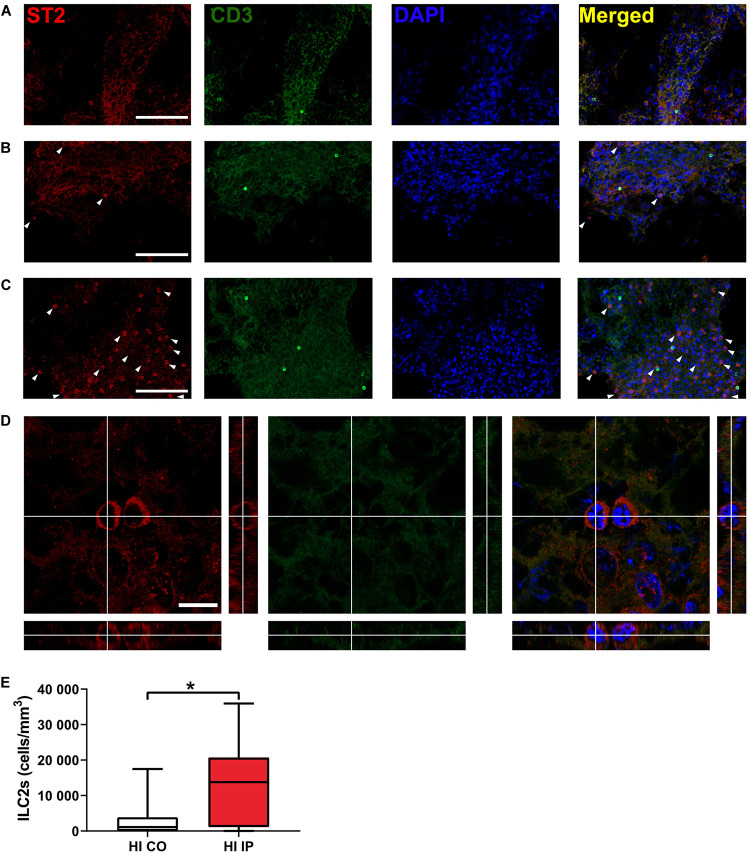
The presence of ILC2s in the meninges in neonatal mice after HI. Representative confocal images of immunofluorescent staining showing the presence of ILC2s (CD3^–^ST2^+^, arrowheads) in the meninges taken from brain hemispheres in a naïve mouse **(A)** and from the contralateral hemisphere **(B)** and ipsilateral hemisphere **(C)** in a mouse at 7 days after HI. **(D)** High magnification orthogonal views of cells stained with ST2. The merged picture shows cytoplasm localization of ST2 surrounding DAPI^+^ nuclei. Scale bars: **(A–C)** 100 μm; **(D)** 10 μm. **(E)** The number density of ILC2-positive cells in the meninges in the ipsilateral hemisphere and contralateral hemispheres after HI. Paired *t*-test was used. *p < 0.05. Abbreviations: HI: hypoxia-ischemia, IP: ipsilateral hemisphere, CO: contralateral hemisphere.

ILC2s are known to be enriched in the meninges compared with the brain parenchyma (Gadani et al., 2017; Russi et al., 2018). We examined the presence of ILC2s at 7 days after HI, when ILC2 accumulation in the brain peaked, using immunofluorescent staining of the meninges compared to naïve mice (Figures 3A–E). ILC2s were found mostly in the meninges of the ipsilateral hemisphere after HI (Figures 3C–E) and only few cells in the meninges contralaterally (Figures 3B,E, *p* = 0.013), or in naïve mice (Figure 3A). In addition, we did not observe ILC2s in the brain parenchyma in either the naïve mice or after HI (data not shown). Because we observed this increase of ILC2s in the injured brain, their role in brain injury after HI was further investigated.

### ILC2-Impaired Neonatal Mice Did Not Respond to IL-33 Stimulation

**FIGURE 4:**
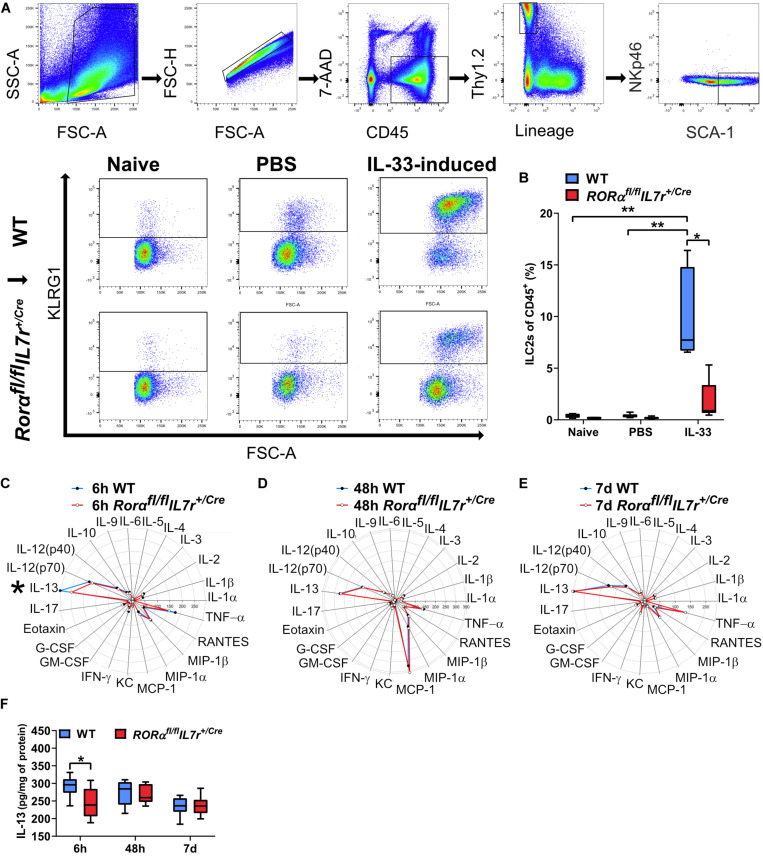
ILC2s in *Rorα^*fl/fl*^IL7r^*Cre*^* mice did not expand after IL-33 stimulation, but their cytokine/chemokine profile in the brain after HI was similar to wild-type mice. **(A)** Representative flow cytometry plots show the gating strategy for IL-33–stimulated ILC2 expansion in the lungs (*n* = 7–9/group). **(B)** IL-33–stimulated ILC2 expansion in wild-type and *Rorα^*fl/fl*^IL7r^*Cre*^* mouse pups. **(C–E)** Radar plots from immunoassays show the cytokine/chemokine changes in the ipsilateral hemisphere from the wild type and *Rorα^*fl/fl*^IL7r^*Cre*^* mouse pups at 6 h **(C)**, 48 h **(D)**, and 7 days **(E)** after HI. **(F)** IL-13 in the ipsilateral hemispheres in the wild type and *Rorα^*fl/fl*^IL7r^*Cre*^* mice at different time points after HI. *p < 0.05, **p < 0.01. Mann–Whitney U-tests were used in **(B)**, and 2-Way ANOVA with Games-Howell post-hoc were used in **(F)**. Abbreviations: HI: hypoxia-ischemia, IP: ipsilateral hemisphere, WT: wild type.

To explore the role of ILC2s in neonatal brain injury, we used the ILC2-deficient mouse strain *Rorα^fl/fl^*IL7r^*Cre*^ (Oliphant et al., 2014). To confirm the ILC2 deficiency in the newborn mice, which has not been investigated previously, we used IL-33, which is known to efficiently induce the expansion and activation of ILC2s (Oliphant et al., 2014; Van Dyken et al., 2014; Besnard et al., 2015; Bartemes et al., 2017), including ILC2s in the CNS (Gadani et al., 2017). We performed IL-33 stimulation experiments to compare the ILC2 response in wild type mice and *Rorα^*fl/fl*^IL7r^*Cre*^* mice (Figures 4A,B, Supplementary Figure 4). Naïve mouse pups without any injection showed similar amount of ILC2s between genotypes, and PBS injections did not evoke ILC2s expansion, as shown by similar amount of ILC2s as in naïve mice (Figure 4B). In contrast, IL-33 triggered massive ILC2 expansion in wild type animals (*n* = 7), but failed to do so in *Rorα^*fl/fl*^IL7r^*Cre*^* mouse pups (*n* = 9, *p* = 0.006) (Figure 4B), thus confirming that the ILC2 expansion in response to IL-33 stimulation in *Rorα^*fl/fl*^IL7r^*Cre*^* mouse pups was significantly impaired.

### ILC2 Impairment Attenuated the IL-13 Increase After HI

To illustrate the inflammatory response in the brain after HI, we performed a Bio-Plex Pro^TM^ Mouse Cytokine 23-plex Assay using brain homogenate from wild type and *Rorα^*fl/fl*^IL7r^*Cre*^* mice. This method allowed the study of 23 different cytokine/chemokines at the same time (Table 1) at 6 h, 48 h, and 7 days after HI (*n* = 7/group) (Figures 4C–F). IL-13 protein levels in the brain were significantly lower at 6 h after HI in *Rorα^*fl/fl*^IL7r^*Cre*^* mice compared with wild-type mice (*p* = 0.046) (Figures 4C,F), while no significant differences were observed for any other cytokine/chemokine between wild type and *Rorα^*fl/fl*^IL7r^*Cre*^* mice (Figures 4C–E) in the ipsilateral hemispheres at any of the time points analyzed.

### ILC2 Impairment Did Not Affect Tissue Loss or the Neuroinflammatory Response in the Neonatal Mouse Brain After HI

**FIGURE 5:**
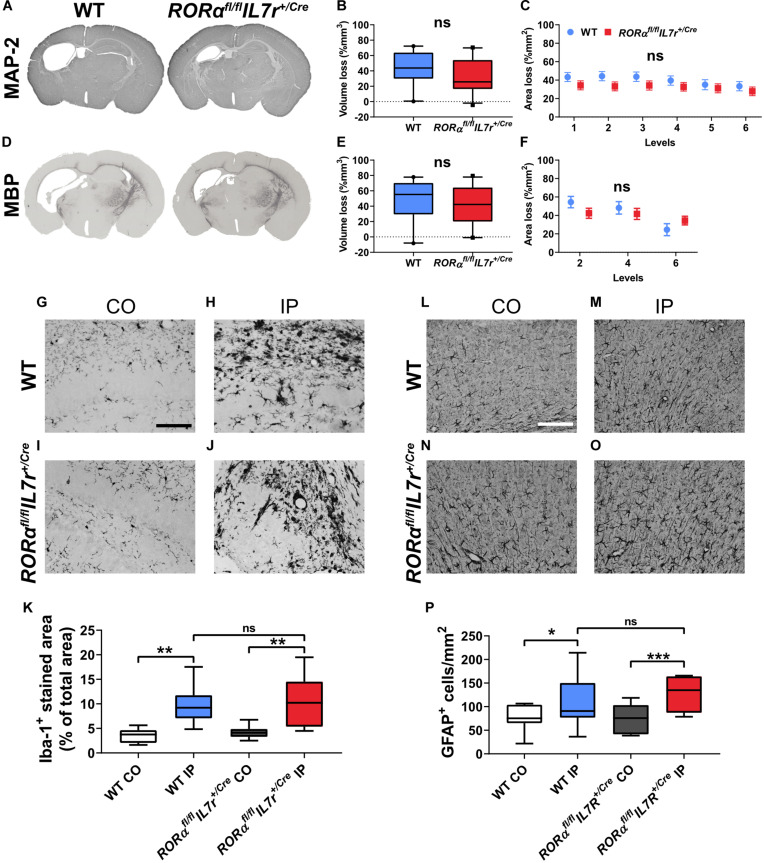
ILC2 impairment did not affect brain tissue loss 7 days after HI. **(A,D)** Representative pictures show immunochemistry staining for MAP-2 **(A)** and MBP **(D)**. Gray matter **(A–C)** in *Rorα^*fl/fl*^IL7r^*Cre*^* mice (*n* = 23) after HI compared to WT littermates (*n* = 21) in terms of brain tissue volume loss **(B)** or area loss per level **(C)**. White matter **(D–F)** tissue loss in the whole hemisphere in terms of tissue volume loss **(E)** or area loss at each level **(F)** in the wild type and *Rorα^*fl/fl*^IL7r^*Cre*^* mice. Representative images **(G–J,L–O)** and quantification **(K,P)** of Iba-1^+^ cells **(G–K)** and GFAP^+^ astrocytes **(L–P)** in the wild type and *Rorα^*fl/fl*^IL7r^*Cre*^* mouse pups at 7 days after HI. Paired *t*-test was used for comparisons between brain hemispheres, and independent *t*-test was used for comparisons between genotypes. Scale bars: 100 μm. Abbreviations: MAP-2: microtubule-associated protein-2, MBP: myelin basic protein; WT: wild type, IP: ipsilateral hemisphere. CO: contralateral hemisphere. *p < 0.05, **p < 0.01, ***p < 0.001.

Next, we investigated the involvement of ILC2s in HI-induced preterm brain injury using the *Rorα^*fl/fl*^IL7r^*Cre*^* mouse strain. Brain injury was evaluated at 7 days after HI for both the gray and white matter. ILC2 impairment did not impact the severity of gray-matter injury either in terms of total tissue loss (Figure 5B) or at different brain levels (Figure 5C) as evaluated by immunohistochemical staining of the neuronal marker MAP-2 (Figures 5A–C). To evaluate white-matter injury, we performed MBP immunohistochemical staining (Figure 5D), and the MBP^+^ white-matter volumes in the whole brain hemisphere (Figure 5E) and areas at different levels (Figure 5F) were measured. Similarly, ILC2 impairment did not affect the tissue loss in white matter in the whole brain hemisphere (Figures 5E,F) or in the subcortical white-matter area (data not shown). Further, no sex difference was noted regarding either the gray or white-matter injury after HI (data not shown).

To explore the neuroinflammatory response in *Rorα^*fl/fl*^IL7r^*Cre*^* after HI insult, we examined the microglia and astrocyte reactivity using immunochemistry staining for the microglia marker Iba-1 and the astrocyte marker GFAP. At 7 days after HI, there were significantly increased staining for microglia (Figures 5G–K) and number of astrocytes (Figures 5L–P) in the ipsilateral hemispheres in both the wild type (microglia: *p* = 0.001, astrocytes: *p* = 0.015) and *Rorα^*fl/fl*^IL7r^*Cre*^* mice (microglia: *p* = 0.003, astrocytes: *p* < 0.001); however, no differences were observed for microglia or astrocytes after HI between the wild type and *Rorα^*fl/fl*^IL7r^*Cre*^* mice in either of the two brain hemispheres.

## Discussion

ILCs and ILC2s have previously been found in the mouse brain and have recently been studied in adult murine models of various brain pathologies (Mair and Becher, 2014; Besnard et al., 2015; Hatfield and Brown, 2015; Russi et al., 2015; Gadani et al., 2017; Russi et al., 2018; Romero-Suarez et al., 2019; Fung et al., 2020). Here we found accumulation of these cells in the meninges after HI injury in neonatal mice, and to our knowledge this is the first study to investigate their presence and function in a neonatal mouse model of brain injury.

Among ILCs, ILC2 is the most abundant subtype in the healthy adult mouse brain (Gadani et al., 2017; Russi et al., 2018). ILC2s have been shown to be increased in tissues in different disease models such as spinal cord injury (Gadani et al., 2017), experimental autoimmune encephalomyelitis (Russi et al., 2015; Russi et al., 2018), experimental cerebral malaria (Besnard et al., 2015), and aging (Fung et al., 2020). In our study, ILCs and ILC2s were present in the normal neonatal mouse brain, and after HI insult ILCs and particularly ILC2s accumulated in the injured brain, which agrees with previous studies (Mair and Becher, 2014; Hatfield and Brown, 2015; Romero-Suarez et al., 2019). This accumulation occurred in a time-dependent manner, and reached the highest level at 7 days after HI. This indicated that ILCs, and especially ILC2s, are stimulated and expanded by HI-induced tissue injury. ILC2s are recognized as tissue-resident cells (Moro et al., 2010; Neill et al., 2010; Price et al., 2010; Molofsky et al., 2013) and are expanded upon IL-33 stimulation in both the peripheral tissue (Oliphant et al., 2014; Van Dyken et al., 2014; Besnard et al., 2015; Bartemes et al., 2017) and the CNS (Gadani et al., 2017). In the current study, we found that neonatal ILC2s in the lung tissue in the wild type mice were also expanded by IL-33 stimulation, which did not occur in the ILC2-deficient mice. Together, this supports the hypothesis that ILC2s in both the peripheral nervous system and CNS are able to respond and expand to either a stimulator like IL-33 and/or stress induced by tissue injury and that they are functional already in early life.

To identify the localization of ILC2 in the neonatal mouse brain, we performed immunofluorescence staining and found that – similar to previous findings (Gadani et al., 2017) – ILC2s in the neonatal mouse brain were resident in the meninges and were seldom found in the brain parenchyma under either normal conditions or after HI-induced brain injury, thus suggesting a role for meningeal immune cells as sentinels for brain-derived alarmins as part of the immune response after brain injury in neonates.

In the current study, among all the cytokine/chemokines examined, we found a small yet significant decrease in expression of IL-13 in the ILC2-deficient mice compared with the wild type mice. IL-13 is considered an anti-inflammatory cytokine and an important modulator of peripheral allergic reactions. In the brain, IL-13 contributes to the death of activated microglia (Yang et al., 2002; Yang et al., 2006) and potentiates the effects of oxidative stress on neurons during neuroinflammation (Park et al., 2009), and both neuroprotective and neurotoxic effects of IL-13 have been proposed. IL-13 has been previously found to be increased both in the serum of term newborns (Orrock et al., 2016; Al-Shargabi et al., 2017; Massaro et al., 2018) and in brain mRNA levels in mice (Albertsson et al., 2014) early after HI injury. In newborns, increased serum IL-13 levels are associated with worse brain injury (Massaro et al., 2018), reduced heart rate variability metrics (Al-Shargabi et al., 2017), and adverse outcome after 24 h of therapeutic hypothermia (Orrock et al., 2016). ILC2s are one of the major producers of IL-13 in response to insults in different tissues (Fallon et al., 2006; Price et al., 2010; Klein Wolterink et al., 2012; Kabata et al., 2018), including in a model of spinal cord injury, where IL-13^+^ cells were found to make up the majority ILC2s (Gadani et al., 2017). The reduction in IL-13 protein levels that we observed here might suggest that ILC2s have a detrimental role. However, in spite of the observed significant increase in ILC2s in the brain after the HI insult, ILC2 impairment did not affect the HI-induced inflammatory responses in the brain or the extent of brain injury after HI in neonatal mice. Similarly, a previous study showed that ILCs infiltrated the brain in an experimental autoimmune encephalomyelitis model but did not affect the severity of injury (Mair and Becher, 2014). Further, we did not observe any sex differences regarding to the degree of injury in the brain of ILC2-deficient mice compared with wild-type mice, although it has been reported previously that the role of ILC2 is sex-dependent under certain circumstances (Bartemes et al., 2017; Russi et al., 2018). Even though ILCs were increased in the CNS after insult, it is possible that there are still not enough ILCs to affect the brain damage development in a substantial manner. Indeed, ILC2s reside mainly in the meninges and choroid plexus, and not in the parenchyma, and their frequencies are generally low in relation to the whole leukocyte population (Hatfield and Brown, 2015; Gadani et al., 2017; Russi et al., 2018; Fung et al., 2020).

ILC2s were the main cell type shown to be responsible for allergic response in a mouse model of lung inflammation (Van Dyken et al., 2014). However, genetic ablation of ILC2s triggered an increase in gamma delta T-cells, thus revealing compensatory mechanisms among innate immune cells (Van Dyken et al., 2014). Another example of cross talk between ILC2s and other innate immune cells was found in a murine model of experimental autoimmune encephalomyelitis where both mast cells and ILC2s were coordinated in the development of brain damage (Russi et al., 2018). We speculate that such cross talk and compensatory mechanisms between ILC2s and other innate immune cells might be among the reasons why ILC2 impairment did not impact HI-induced brain injury in neonatal mice.

In addition, the role of other ILCs such as ILC1 and ILC3 remains an important topic for future study. The lymphoid tissue inducer cells comprise the ILC3 population. They present as early as the embryonic stage and play an important role in intestinal homeostasis after birth (Eberl, 2012), this might indicate the importance of ILC3s in the immune response in the perinatal period during development. In the current study, we did not observe any obvious increase of ILC1 and ILC3 in the brain after HI (data not shown), which might be due to the time points at which ILC1 and ILC3 levels were assessed. Furthermore, whether or not there are compensatory changes in ILC1 and ILC3 that contribute to the lack of an effect from ILC2 deficiency also needs to be explored in the future.

Exploration of the neuroinflammatory response after HI by immunohistochemistry revealed increased numbers of astrocytes and staining for microglia in the ipsilateral hemisphere at 7 days after HI in both ILC2-deficient and wild-type mice, and this agreed with previous findings (Doverhag et al., 2010; Bonestroo et al., 2013). However, there was no significant difference in astrocytes and microglia in either of the brain hemispheres between wild type and ILC2-deficient mice, and these results are thus in line with our findings that no significant differences were observed for cytokines/chemokines between wild type and ILC2-deficient mice apart from IL-13 and that there were no differences in the severity of brain injury between the two mouse genotypes.

We found increased accumulation of ILC2s in the injured brain after HI, but ILC2 deficiency did not affect the severity of the injury. We found no differences in the neuroinflammatory response after HI between ILC2-deficient and wild-type mice that might partly explain the lack of effect of ILC2 deficiency on brain injury. However, the in-depth molecular mechanisms behind these findings are not known. A limitation of the study is that we only assessed effect of ILC2 deficiency on brain injury at 7 days after HI. We cannot exclude the possibility that ILC2 deficiency might have an effect at a later time point after HI.

In conclusion, ILCs and ILC2s accumulate in the injured brain after HI insult in the neonatal mouse brain. However, ILC2s did not affect the major inflammatory response in the brain and did not contribute to the development of brain damage in this mouse model of preterm brain injury.

## Data Availability Statement

All datasets generated for this study are included in the article/Supplementary Material.

## Ethics Statement

The animal study was reviewed and approved by the Regional Animal Ethical Committee of Gothenburg (ethical permit numbers: 58/2016 and 2042/18).

## Author Contributions

XW conceptualized the study. AZ, ER-F, and AN designed the experiments and performed the flow cytometry experiments. AZ and ER-F performed the HI model. AZ performed the protein assays, immunohistochemistry, and immunofluorescence staining. AZ and TC performed fluorescent imaging and analyzed the immunofluorescence data. AZ and MA performed the statistical analysis. AZ and XW drafted the manuscript. All authors contributed to data interpretation and critical revision of the manuscript and approved the submitted version.

## Conflict of Interest

The authors declare that the research was conducted in the absence of any commercial or financial relationships that could be construed as a potential conflict of interest.

## Footnotes

1 https://imagej.nih.gov/ij/, 1997–2016

## Supplementary Material

FIGURE S1 Representative images show the positive controls (lung tissue, **A**), and negative controls omitting the primary antibodies for the immunofluorescent staining of ST2 and CD3 for lung tissue **(B)** and meninges **(C)**. Arrow head: ST2+ ILC2s, star: CD3+ cells. Scale bars: 50 μm.

FIGURE S2 Representative flow cytometry plots for fluorescence-minus-one controls (FMO) used in flow cytometry experiments.

FIGURE S3 **(A)** Representative flow cytometry plots for intracellular staining of GAGA-3. **(B)** The frequency of Lin^–^CD45^+^GATA3^+^ ILC2s at 7 days post HI in the neonatal wild-type mice. Paired *t*-test was used in B for comparison between hemispheres. ^∗^*p* < 0.05.

FIGURE S4 Representative flow cytometry plots for fluorescence-minus-one controls (FMO) used in flow cytometry experiments for the lung tissue IL-33 experiments.
