# Supplementary material for: Type 2 Innate Lymphoid Cells Accumulate in the Brain After Hypoxia-Ischemia but Do Not Contribute to the Development of Preterm Brain Injury
Source: Front Cell Neurosci. 2020 Aug 7;14:249. doi: 10.3389/fncel.2020.00249 (PMC7426829; doi:10.3389/fncel.2020.00249)
Supplement: FIGURE S1 — Representative images show the positive controls (lung tissue, A), and negative controls omitting the primary antibodies for the immunofluorescent staining of ST2 and CD3 for lung tissue (B) and meninges (C). Arrow head: ST2+ ILC2s, star: CD3+ cells. Scale bars: 50 μm. [file Data_Sheet_1.pdf]

Supplementary Material

Type 2 innate lymphoid cells accumulate in the brain after hypoxia-ischemia but do not contribute to the development of preterm brain injury

Aura Zelco[1], Eridan Rocha-Ferreira[2], Arshed Nazmi[1], Maryam Ardalan[1], Tetyana Chumak[1] Gisela Nilsson[1], Henrik Hagberg[2], Carina Mallard[1], Xiaoyang Wang[1,3*]

[1]Institute of Neuroscience and Physiology, Department of Physiology, Sahlgrenska Academy, University of Gothenburg, Gothenburg, Sweden

[2]Centre of Perinatal Medicine & Health, Institute of Clinical Sciences, Sahlgrenska Academy, University of Gothenburg, Gothenburg, Sweden

[3]Henan Key Laboratory of Child Brain Injury, Institute of Neuroscience and Third Affiliated Hospital of Zhengzhou University, Zhengzhou 450052, China

*** Correspondence:**

Xiaoyang Wang, MD, PhD, Centre for Perinatal Medicine & Health, Department of Neuroscience and Physiology, University of Gothenburg, Box 432, SE-405 30 Gothenburg, Sweden, Tel: +46 31 786 3260, Fax +46 31 786 35 12; Email: xiaoyang.wang@fysiologi.gu.se

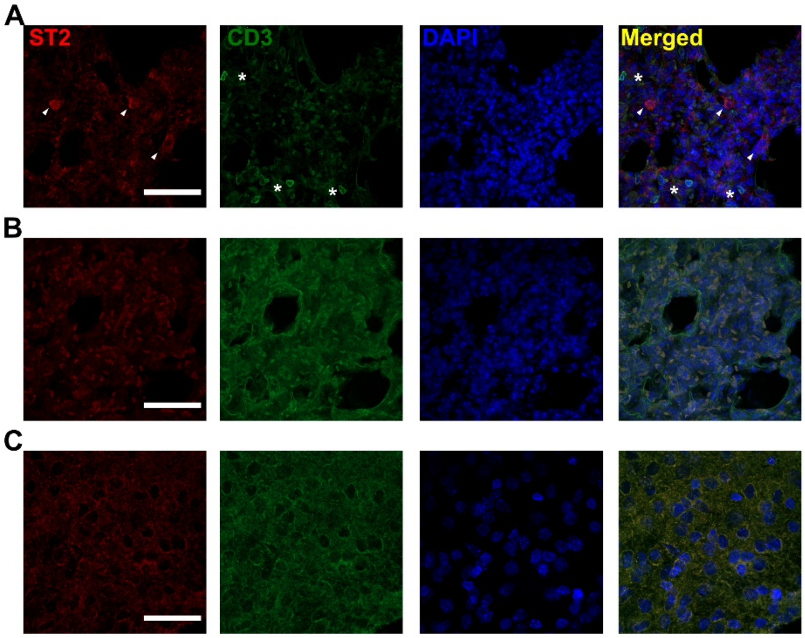

Supplementary Figure 1. Representative images show the positive controls (lung tissue, A), and negative controls omitting the primary antibodies for the immunofluorescent staining of ST2 and CD3 for lung tissue (B) and meninges (C). Arrow head: ST2+ ILC2s, star: CD3+ cells. Scale bars: 50 µm.

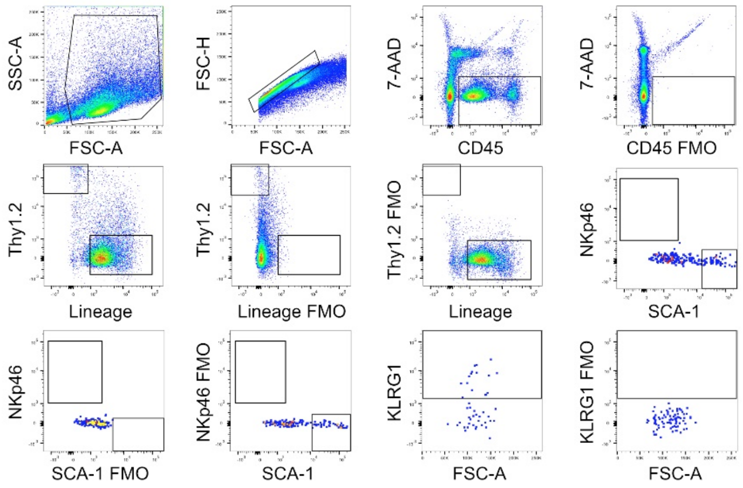

Supplementary Figure 2. . Representative flow cytometry plots for fluorescence-minus-one controls (FMO) used in flow cytometry experiments.

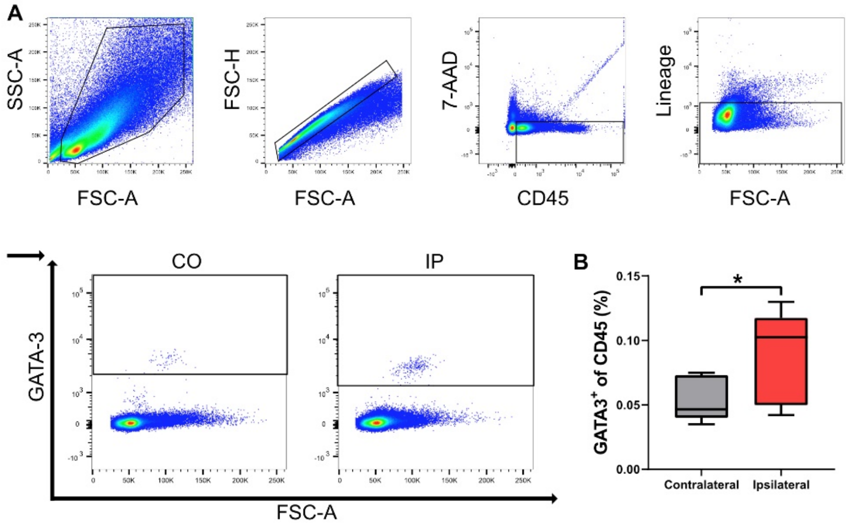

Supplementary Figure 3. (A) Representative flow cytometry plots for intracellular staining of GAGA-3. (B) The frequency of Lin^{-}CD45^{+}GATA3^{+} ILC2s at 7 d post HI in the neonatal wild-type mice. Paired t-test was used in B for comparison between hemispheres. *, p<0.05.

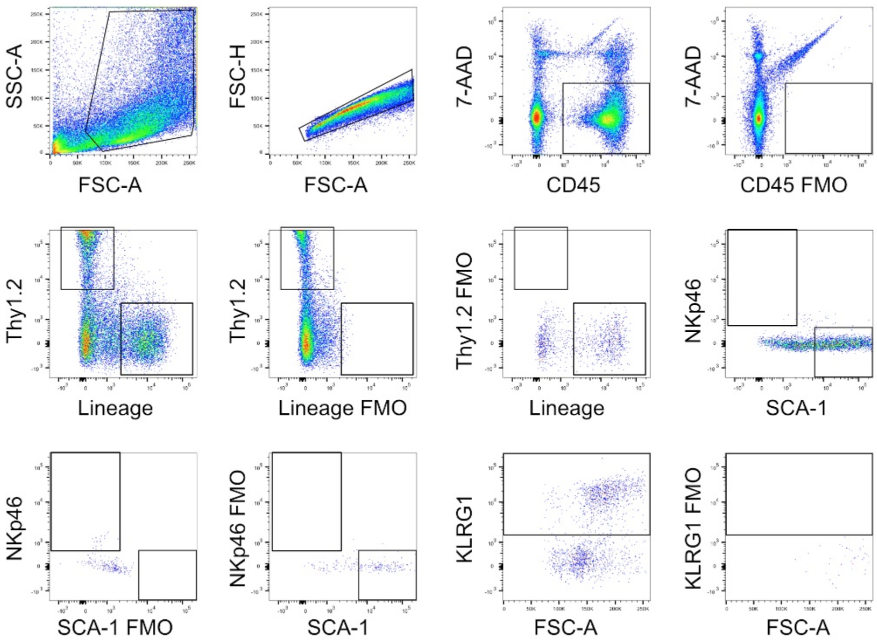

Supplementary Figure 4. Representative flow cytometry plots for fluorescence-minus-one controls (FMO) used in flow cytometry experiments for the lung tissue IL-33 experiments.
